# Prevalence and trends in overactive bladder among men in the United States, 2005–2020

**DOI:** 10.1038/s41598-024-66758-8

**Published:** 2024-07-15

**Authors:** Yu Cheng, Tao Chen, Guanghao Zheng, Zhen Song, Gan Zhang, Xuepeng Rao, Tao Zeng, Changfei Yuan

**Affiliations:** The Second Affiliated Hospital of Nanchang University, Jiangxi Medical College, Nanchang University, No. 1, Minde Road, Nanchang, 330006 Jiangxi China

**Keywords:** Overactive bladder, Prevalence, National Health and Nutrition Examination Survey, Risk factors, Urgency incontinence, Nocturia, Men, Bladder, Risk factors

## Abstract

The purpose of present study was to examine the current prevalence and recent trends of overactive bladder (OAB) among US adult men and examine the correlations between OAB and several potential risk factors. The study used the nationally representative data between 2005 and 2020 from the National Health and Nutrition Examination Survey in the US. A total of 18,386 participants aged ≥ 20 years were included in the study. We divided the data into three groups: 2005–2008, 2009–2014 and 2015–2020 to investigate the trends in OAB prevalence. The weighted prevalence and corresponding 95% confidence intervals (CI) of OAB were calculated. The differences (95% CI) in prevalence between the surveys were calculated and multivariate-adjusted weighted logistic regression analysis was performed to determine the correlates of OAB. Among all US adult men, the overall prevalence of OAB increased slightly from 11.3% in 2005–2008 to 11.7% in 2009–2014 and significantly increased to 14.5% in 2015–2020 (difference, 3.2% [95% CI (1.9–4.4%)]; *P* < 0.05). Increases in OAB prevalence especially concentrated on those who were 40–59 years, non-Hispanic White, non-Hispanic Black and those who were overweight and obese. Older age, non-Hispanic Black, lower educational level and family poverty ratio, diabetes, depression, sleep disorder, other chronic comorbidities, less intense recreational activity, poorer health condition and unsafe food were independent risk factors of OAB. The contemporary prevalence of OAB was high, affecting 14.5% US men and the estimated overall prevalence significantly increased from 2005 to 2020. Therefore, future research should be focused to prevent and remedy this growing socioeconomic and individually troublesome malady.

## Introduction

Overactive bladder (OAB) is a widespread condition that affects millions of individuals globally, with a high prevalence among men and women^1^. It can have significant impact on physical and psychological health, and quality of life^2^. Additionally, OAB caused a substantial economic burden, with estimated costs of 7 billion euros in Europe and 66 billion USD in the United States (US) annually, these costs include expenses related to urgency urinary incontinence (UUI) and nursing home admissions^3^. As the population aged and living standards improved, the financial burden is projected to increase in the future.

OAB is a condition characterized by urinary urgency, increased frequency during the day and night (nocturia), accompanied by UUI or not, and without urinary tract infection^4^. The overall prevalence of OAB varies across different countries and studies. In the EPIC study performed in five western countries, the prevalence was estimated to be 11.8%^1^. An OAB-POLL study reported the prevalence was 8% in men and 20% in women in the US^5^. The overall prevalence of OAB in Asia was 20.8%, and the prevalence of OAB in China was 23.9%^6^, while in Austria it was 16.8%^7^ and in Europe and Canada it was 10.8% and 12.8% in men and women respectively^1^. The difference in OAB prevalence rate may be related to the geographic distribution, method of epidemiological investigation, study designs, different OAB definitions, response rates, and study population. Although recent epidemiology of OAB has focused on men and women^8,9^, the current incidence and recent trends in male OAB are unclear.

The correlated factors and pathogenesis of OAB remain uncertain, age, socioeconomic status, lifestyles, nutritional status and comorbidities may all be associated with OAB^10^. To address these gaps, we used nationally representative data from the National Health and Nutrition Examination Survey (NHANES) to evaluate the contemporary prevalence and trends in OAB from 2005 to 2020 among US men and to further determine the associations between sociodemographic features, BMI, chronic comorbidities, lifestyles and OAB.

## Materials and methods

### Study design

The NHANES is a program of surveys conducted by the National Center for Health Statistics (NCHS) to collect health-related data from the civilian noninstitutionalized population in the US. These surveys use a complex and multistage probability sample design to establish a representative sample of the population. The aim is to obtain comprehensive information on health and nutrition status through interviews and examinations. The detailed information of study design, protocol, and data collection for NHANES are described in existing publications^11^. From 1999 to March 2020, there were a total of ten cycles for NHANES surveys, consisting of nine 2-year cycles spanning from 1999 to 2016 and one combined cycle from 2017 to March 2020, which was impacted by the COVID-2019 pandemic^12^. For this study, the NHANES protocol obtained approval from the NCHS Research Ethics Review Board, and all participants provided written informed consent. The present study included adult men aged ≥ 20 years and who had complete data for OAB associated symptoms including UUI and nocturia from the 2005–2006 cycle through the 2017–2020 cycle, the unweighted total male response rates ranged from 49.7 to 80.0% for the interviewed samples and from 45.6 to 77.1% for the examined samples^13^.

### Assessment and definitions of OAB

The data on OAB associated symptoms including UUI and nocturia were collected by using Kidney Conditions-Urology (KIQ_U) questionnaire in the mobile examination center (MEC). Two questions were asked to assess the severity of UUI: 1. “During the past 12 months, have you leaked or lost control of even a small amount of urine with an urge or pressure to urinate and you couldn’t get to the toilet fast enough?” 2. “How frequently does this occur?” And another question was asked to assess the severity of nocturia: “During the past 30 days, how many times per night did you most typically get up to urinate, from the time you went to bed at night until the time you got up in the morning?”.

To further identify OAB, we used the established OABSS questionnaire developed by Blaivas et al.^14^ The method for conversion of NHANES symptom frequencies to OABSS are shown in Table 1. Finally, we combined the UUI score and nocturia score and total score of ≥ 3 indicated a diagnosis of OAB disorder.

**Table 1:** Conversion of NHANES symptom frequencies to OABSS.

NHANES symptom frequencies	OABSS
*UUI*	*UUI score*
Never	0
Less than once a month	1
A few times a month	1
A few times a week	2
Every day or night	3
*Nocturia*	*Nocturia score*
0	0
1	1
2	2
3	3
4	3
5 or more	3

NHANES, national health and nutrition examination survey; OABSS, overactive bladder symptom score; UUI, urgency urinary incontinence.

### Correlates of OAB

We extracted data on age (20–39 years, 40–59 years and ≥ 60 years), race/ethnicity (Hispanic, non-Hispanic White, non-Hispanic Black, non-Hispanic Asian and other), education (< high school, high school and > high school), family poverty ratio (a ratio of family income to poverty threshold; < 1.3, 1.3–3.5 and ≥ 3.5), body mass index (BMI; < 25 kg/m^2^, 25–30 kg/m^2^, ≥ 30 kg/m^2^), smoking (yes, no), hypertension (yes, no), diabetes (yes, no), depression (yes, no), sleep time (yes, no), sleep disorder (yes, no), chronic conditions (including asthma, arthritis, congestive heart failure, coronary heart disease, angina/angina pectoris, heart attack, stroke, thyroid problem, any liver condition and cancer), recreational activity (sports, fitness, or recreational activities; mild, moderate and vigorous), healthy diet (yes, no), general health condition (good, not good), food security (safe, not safe) and health insurance (yes, no).

### Statistical analysis

Statistical analysis was performed with *R* version 4.3.1. Data from NHANES 2005–2006 to 2017–2020 were divided into three surveys: 2005–2008, 2009–2014 and 2015–2020. Descriptive statistics were conducted to show the demographic characteristics and other participants’ features in each survey. Estimates on weighted overall prevalence (95% confidence interval (CI)) of OAB were calculated in each survey and were further calculated by age group, race/ethnicity group and BMI group. The differences (95% CI) of prevalence between the surveys were calculated and were considered to be statistically significant if χ^2^ test had a *P* value of less than 0.05. To perform the multiple logistic regression analysis, we tested the multicollinearity of independent variables and found no multicollinearity in these variables. In addition, the sample size was enough to conduct the multiple logistic regression analysis. Thus, weighted logistic regressions were used to explore the correlates of OAB by incorporating age, race/ethnicity, education, family poverty ratio, body mass index, smoking, hypertension, diabetes, depression, sleep time, sleep disorder, chronic conditions, recreational activity, diet, health condition, food security and health insurance. All statistical tests were 2 sided, with *P* < 0.05 considered statistically significant.

## Results

### Participant characteristics

Of the 76,496 individuals who participated in NHANES from 2005 to 2020, we excluded 33,084 participants who were younger than 20 years, 22,385 who were women, and 2641 who had incomplete information of UUI and/or nocturia. The final study population included 18,386 male adults aged ≥ 20 years and who had complete data. There was no significant difference of the most variables including age and race/ethnicity composition between the three surveys. Detailed data on demographic characteristics and other participants’ features in each survey were shown in Table 2.

**Table 2:** Baseline characteristics of the study population in the NHANES, 2005 to 2020.

Characteristic	2005–2008	2009–2014	2015–2020	*P* value^a^
Number	4652	7429	6305	
Weighted (*N*)	188,491,662	297,519,614	214,838,237	
Age, y				> 0.05
20–39	39.2	37.5	36.9	
40–59	39.3	37.9	35.6	
≥ 60	21.5	24.6	27.5	
Race/ethnicity				> 0.05
Mexican American	9.0	9.1	8.9	
Other Hispanic	3.9	5.5	7.0	
Non-Hispanic White	71.6	68.1	64.4	
Non-Hispanic Black	10.3	10.2	10.4	
Other^b^	5.2	7.1	9.2	
Education				< 0.05
< High school	19.5	16.8	13.3	
High school	25.7	22.8	25.3	
> High school	54.7	60.4	61.4	
Family poverty ratio				> 0.05
< 1.3	17.1	21.5	17.7	
1.3–3.5	36.0	34.4	35.3	
≥ 3.5	46.9	44.2	47.0	
BMI, kg/m^2^				> 0.05
< 25	27.3	26.4	24.0	
25–30	39.8	38.5	36.1	
≥ 30	32.9	35.1	39.9	
Smoke				> 0.05
Current	26.9	21.9	19.7	
Former	28.8	28.2	31.1	
Never	44.3	49.9	49.2	
Hypertension				> 0.05
Yes	29.2	32.2	33.1	
No	70.8	67.8	66.9	
Diabetes				< 0.05
Yes	7.8	9.6	13.0	
No	92.2	90.4	87.0	
Depression				> 0.05
Yes	5.0	5.7	5.9	
No	95.0	94.3	94.1	
Sleep time, h				
< 7	38.8	37.2	25.6	
≥ 7	61.2	62.7	74.4	
Sleep disorder				> 0.05
Yes	20.1	22.9	27.6	
No	79.9	77.1	72.4	
Chronic diseases^c^				> 0.05
Any	41.1	41.5	44.7	
No	58.9	58.5	55.3	
Recreational activity				> 0.05
Mild	38.2	43.8	42.4	
Moderate	26.7	26.5	23.7	
Vigorous	35.1	29.7	33.8	
Health diet				> 0.05
Yes	71.8	73.3	69.4	
No	28.2	26.7	30.6	
Health condition				> 0.05
Good	83.5	83.3	82.6	
Not good	16.5	16.7	17.4	
Food security				< 0.05
Yes	90.5	86.2	83.7	
No	9.5	13.8	16.3	
Health insurance				> 0.05
Yes	77.8	78.3	84.4	
No	22.2	21.7	15.6	

Data were expressed as %. NHANES National Health and Nutrition Examination Survey, BMI body mass index.

^a^All estimates were weighted to be nationally representative. No. of participants for some variables may not sum up to equal the unweighted and weighted number due to missing data.

^b^“Other” includes race and ethnicity other than non-Hispanic White, non-Hispanic Black, and Hispanic, including multiracial.

^c^Chronic diseases included asthma, arthritis, congestive heart failure, coronary heart disease, angina/angina pectoris, heart attack, stroke, thyroid problem, any liver condition and cancer.

### Prevalence and trends of OAB

**Figure 1:**
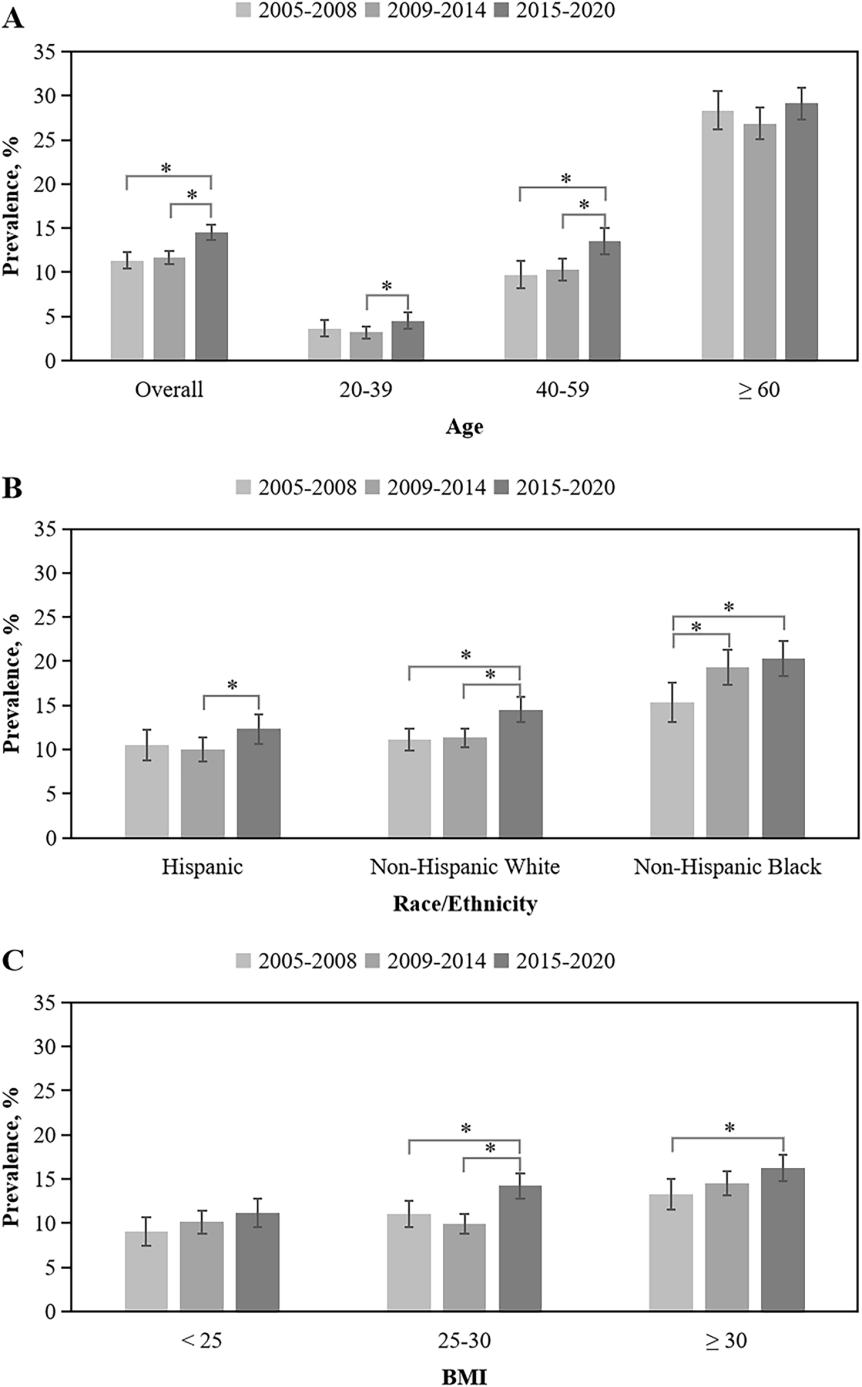
Trends in OAB prevalence by age (**A**), race/ethnicity (**B**) and BMI (**C**). *P < 0.05, BMI body mass index, OAB overactive bladder.

The overall prevalence of OAB increased slight from 11.3% in 2005–2008 to 11.7% in 2009–2014 and increased significantly to 14.5% in 2015–2020 (difference, 3.2% [95% CI 1.9–4.4%)]; *P* < 0.05) (Table 3 and Fig. 1A). A similar increasing pattern was observed for men aged 40–59 years from 9.7% to 13.5% (difference, 3.8% [95% CI 1.7–5.9%)]; *P* < 0.05). For those aged 20–39 years, the prevalence had a slightly decrease from 3.6% to 3.2%, but significantly rose to 4.5% (*P* < 0.05); yet the prevalence was stable for those aged ≥ 60 years (*P* > 0.05) from 2005–2008 to 2015–2020. By race/ethnicity group, the prevalence of OAB increased significantly from 11.1% to 14.5% (difference, 3.4% [95% CI 1.5–5.3%)]; *P* < 0.05) for non-Hispanic White and from 15.4% to 20.3% (difference, 4.9% [95% CI 1.9–8.0%]; *P* < 0.05) for non-Hispanic Black from 2005–2008 to 2015–2020. For Hispanic, the prevalence had a slightly decrease from 10.5% to 10.0%, but significantly rose to 12.3% (*P* < 0.05) (Fig. 1B). By BMI group, the prevalence of OAB for overweight men increased from 11.0% in 2005–2008 to 14.2% in 2015–2020 (difference, 3.2% [95% CI (1.0–5.4%]; *P* < 0.05), and increased from 13.2% to 16.3% (difference, 3.1% [95% CI (0.8–5.3%]; *P* < 0.05) for obese men, while the prevalence was stable for those with a BMI < 25 kg/m^2^ (Fig. 1C). Detailed data were shown in Table 3.

**Table 3:** Weighted trends in OAB prevalence among US men, NHANES 2005 to 2020.

	2005–2008	Difference^b^	2009–2014	Difference^c^	2015–2020	Total difference^d^
Overall^a^	11.3 (10.4–12.3)	0.4 (− 0.8–1.5)	11.7 (11.0–12.4)	2.8 (1.7–3.9)*	14.5 (13.6–15.4)	3.2 (1.9–4.4)*
Age, year						
20–39	3.6 (2.7–4.6)	− 0.4 (− 1.5–0.7)	3.2 (2.5–3.9)	1.3 (0.2–2.5)*	4.5 (3.6–5.4)	0.9 (− 0.4–2.2)
40–59	9.7 (8.2–11.2)	0.6 (− 1.4–2.5)	10.3 (9.1–11.5)	3.2 (1.3–5.1)*	13.5 (12.0–15.0)	3.8 (1.7–5.9)*
≥ 60	28.3 (26.1–30.5)	− 1.4 (− 4.3–1.3)	26.9 (25.1–28.6)	2.2 (− 0.3–4.8)	29.1 (27.3–30.9)	0.8 (− 2.1–3.6)
Race/Ethnicity						
Hispanic	10.5 (8.7–12.2)	− 0.5 (− 2.7–1.8)	10.0 (8.6–11.4)	2.3 (0.1–4.5)*	12.3 (10.7–14.0)	1.8 (− 0.6–4.2)
Non-Hispanic White	11.1 (9.9–12.4)	0.2 (− 1.5–1.9)	11.3 (10.2–12.4)	3.2 (1.4–5.0)*	14.5 (13.1–16.0)	3.4 (1.5–5.3)*
Non-Hispanic Black	15.4 (13.1–17.6)	3.9 (1.0–6.9)*	19.3 (17.4–21.3)	1.0 (− 1.8–3.8)	20.3 (18.3–22.3)	4.9 (1.9–8.0)*
BMI, kg/m^2^						
< 25	9.1 (7.5–10.7)	1.0 (− 1.0–3.1)	10.1 (8.8–11.4)	1.1 (− 1.0–3.1)	11.2 (9.6–12.7)	2.1 (− 0.1–4.3)
25–30	11.0 (9.6–12.5)	− 1.1 (− 3.0–0.7)	9.9 (8.8–11.0)	4.3 (2.5–6.2)*	14.2 (12.8–15.7)	3.2 (1.0–5.4)*
≥ 30	13.2 (11.5–15.0)	1.3 (− 0.9–3.5)	14.5 (13.1–15.9)	1.8 (− 0.3–3.8)	16.3 (14.8–17.8)	3.1 (0.8–5.3)*

Data were expressed as % (95% CI). OAB, overactive bladder; NHANES, National Health and Nutrition Examination Survey; BMI, body mass index; CI, confidence interval.

^a^All estimates were weighted to be nationally representative.

^b^The difference of OAB prevalence between 2005–2008 and 2009–2014**,** a negative value indicates a decrease in the prevalence between the two time periods .

^c^The difference of OAB prevalence between 2009–2014 and 2015–2020**,** a negative value indicates a decrease in the prevalence between the two time periods.

^d^Total difference means the difference between 2015–2020 and 2005–2008.

*P < 0.05.

### Correlates of OAB

In multivariate logistic regression analysis, the prevalence of OAB was higher for those aged ≥ 60 years (OR 7.21; 95% CI 5.75–9.03) and those aged 40–59 years (OR 2.63; 95% CI 2.20–3.14) when compared with men aged 20–39 years (Table 4 and Fig. 1A). Hispanic (OR 1.07; 95% CI 0.91–1.26) and non-Hispanic Black men (OR 1.91; 95% CI 1.62–2.24) had a higher prevalence of OAB than non-Hispanic White men (Fig. 1B). In addition, for those who had diabetes (OR 1.54; 95% CI 1.32–1.80), depression (OR 2.44; 95% CI 1.97–3.04), sleep disorder (OR 1.27; 95% CI 1.07–1.51), chronic conditions (OR 1.67; 95% CI 1.43–1.93), mild recreational activity (OR 1.31; 95% CI 1.04–1.65) or moderate recreational activity (OR 1.35; 95% CI 1.05–1.73), poorer health condition (OR 1.34; 95% CI 1.14–1.59) and unsafe food (OR 1.31; 95% CI 1.09–1.57) had a significantly higher prevalence of OAB. For those who had high school (OR 0.80; 95% CI 0.65–0.97) or higher than high school educational level (OR 0.65; 95% CI 0.55–0.77), family poverty ratio between 1.3 and 3.5 (OR 0.86; 95% CI 0.73–1.00) or ≥ 3.5 (OR 0.74; 95% CI 0.61–0.90) had a significantly lower prevalence of OAB (Table 4).

**Table 4:** Weighted logistic regression models of OAB among men, NHANES 2005 to 2020.

Characteristics	OR (95% CI)	*P*^a^
Age, y		< 0.001
20–39	1 (Reference)	
40–59	2.63 (2.20, 3.14)	
≥ 60	7.21 (5.75, 9.03)	
Race/ethnicity		< 0.001
Non-Hispanic White	1 (Reference)	
Non-Hispanic Black	1.91 (1.62, 2.24)	
Hispanic	1.07 (0.91, 1.26)	
Education		< 0.001
< High school	1 (Reference)	
High school	0.80 (0.65, 0.97)	
> High school	0.65 (0.55, 0.77)	
Family poverty ratio		0.010
< 1.3	1 (Reference)	
1.3–3.5	0.86 (0.73, 1.00)	
≥ 3.5	0.74 (0.61, 0.90)	
BMI, kg/m^2^		0.2
≤ 25	1 (Reference)	
25–30	1.02 (0.87, 1.21)	
≥ 30	1.19 (0.99, 1.45)	
Smoke		0.6
Never	1 (Reference)	
Former	1.03 (0.86, 1.23)	
Current	1.10 (0.90, 1.34)	
Hypertension		0.2
No	1 (Reference)	
Yes	1.09 (0.93, 1.28)	
Diabetes		< 0.001
No	1 (Reference)	
Yes	1.54 (1.32, 1.80)	
Depression		< 0.001
No	1 (Reference)	
Yes	2.44 (1.97, 3.04)	
Sleep time, h		0.14
≥ 7	1 (Reference)	
< 7	0.90 (0.78, 1.04)	
Sleep disorder		0.006
No	1 (Reference)	
Yes	1.27 (1.07, 1.51)	
Any chronic disease^b^		< 0.001
No	1 (Reference)	
Yes	1.67 (1.43, 1.93)	
Recreational activity		0.049
Vigorous	1 (Reference)	
Moderate	1.35 (1.05, 1.73)	
Mild	1.31 (1.04, 1.65)	
Healthy diet		0.4
Yes	1 (Reference)	
No	1.06 (0.92, 1.22)	
Health condition		< 0.001
Good	1 (Reference)	
Not good	1.34 (1.14, 1.59)	
Food security		0.003
Yes	1 (Reference)	
No	1.31 (1.09, 1.57)	
Health insurance		0.8
Yes	1 (Reference)	
No	0.98 (0.79, 1.21)	

OAB, overactive bladder; NHANES, National Health and Nutrition Examination Survey; OR, odds ratio; CI, confidence interval; BMI, body mass index.

^a^All estimates were weighted to be nationally representative.

^b^Chronic diseases included asthma, arthritis, congestive heart failure, coronary heart disease, angina/angina pectoris, heart attack, stroke, thyroid problem, any liver condition and cancer.

## Discussion

Data on the epidemiology of male OAB are scarce. About two decades ago, the National Overactive Bladder Evaluation (NOBLE) program was performed to investigate the overall prevalence and burden of OAB in the US and reported an overall prevalence of 16.0% for US men in 2002^15^ using clinically validated computer-assisted telephone interview (CATI) questionnaire which was different from OABSS. Another study published in 2013 reported the prevalence of OAB defined by the presence of urinary urgency ≥ ‘‘often,’’ and/or the presence of UUI was around 8% in US men^5^. The difference in OAB prevalence between our study and previous studies may be related to the method of epidemiological investigation, study designs, different OAB definitions, response rates, and study population. The contemporary prevalence and recent trends in male OAB were unknown. The present study, for the first time, systematically evaluated nearly 20-year trend in OAB among US men. Although two prior studies that revealed the overall incidence of UUI increased significantly from 2005 to 2018^16^ and the overall incidence of nocturia increased significantly from 2005 to 2016^17^ in US men, the authors did not evaluate the prevalence of OAB, thus the increasing trends in UUI and nocturia may be attributed to the increasing OAB prevalence.

The reason and mechanism of increasing OAB prevalence remained unclear, it may not because of the increase in aging population, as we did not observe the increasing trend of OAB prevalence in those aged ≥ 60 years. Additionally, although we found a significant increase in non-Hispanic Black men, the proportion of Black men did not increase across the three surveys. Although obese persons seemed to be increasing and an increasing trend was observed in obese men, we failed to demonstrate an independent association of obesity and OAB. Nevertheless, neurological disorders, particularly Parkinson disease and multiple sclerosis, and antidepressant use are potential causes of OAB, and are increasing in the US in recent years^18–20^. Therefore, future study should be focused to explore the reasons why OAB prevalence increased.

The prevalence and trends of OAB were found to be associated with some sociodemographic features. Non-Hispanic Black men had a higher prevalence of OAB, this was consistent with the results of a racially diverse population study^21^. Moreover, a significant increase trend was observed in non-Hispanic Black men in our study. However, the specific reasons for racial differences in OAB remained unclear, they were likely multifactorial. OAB is commonly found in men diagnosed with benign prostatic hyperplasia (BPH) and/or an enlarged prostate. The risk of developing BPH is significantly higher among non-Hispanic Black and Hispanic men compared to non-Hispanic White men^22,23^. Furthermore, racial minorities in the US tend to have lower educational levels, engage in physically demanding jobs, earn lower incomes, and experience higher stress levels. These factors have been associated with an increased risk of urinary system diseases^24,25^. Additionally, the reason that a higher educational level and family poverty ratio had a lower prevalence of OAB^6,10,15^ may be attributed to the fact those people tend to have better health-seeking behaviors and adopt healthier lifestyles. Conversely, individuals with lower education and income levels may have a higher prevalence of smoking, poor diet, increased physical labor, and exposure to toxins, which increases their susceptibility to developing OAB.

Chronic comorbidities, diabetes, stroke or cancer, were all associated with a higher prevalence with OAB, probably by compromising pelvic floor vascular, nerve and muscle function^26–28^. Stroke, diabetes and cancer may cause neurological conditions such as central nerve system injury and diabetic autonomic neuropathy, which may lead to the occurrence of OAB^29^. Additionally, depression and sleep disorder were associated with a higher prevalence of OAB in our study. Previous studies demonstrated that OAB or its associated symptoms such as nocturia may result in poor sleep and sleep apnea^30,31^, and mental health conditions such as anxiety and depression may influence the natural history of OAB in US veterans^32^. In fact, sleep or mental problems and OAB may be causal to each other, OAB can obviously affect mental health and sleep quality and sleep disorder or mental problems such as anxiety and depression, in turn, affect and aggravate the condition of OAB, which may be related to the dysregulation of bladder function by numerous neural pathways. Additionally, this analysis found that those who had a vigorous recreational activity, good health condition and good food security had a lower risk of experiencing OAB. Therefore, these healthy lifestyles are recommended in daily life to prevent the occurrence of OAB and relieve or manage OAB.

The NHANES design constituted the principal strengths of this study. NHANES employed a sophisticated multistage, probability-based sampling procedure to enroll a sample that properly represented the overall United States population. Furthermore, standardized protocols ensured the quality of data collection in NHANES. However, this study had some limitations, such as the fact that confirmation of OAB relied on self-reported information with no comprehensive clinical examination, including physical assessment, urine analysis, ultrasound, or urodynamic testing. Additionally, recall biases could have influenced the self-reported data used in the study. Due to the cross-sectional nature of the study, it was not possible to capture newly developed cases of OAB or assess the duration of OAB. Finally, the noninstitutionalized nature of participants included in the study implied that the actual prevalence of OAB may have been underestimated since individuals in hospitals or nursing homes were not represented. However, the present study provided crucial observations of contemporary epidemiology of OAB, and disparities of OAB in sociodemographic, comorbidities and lifestyles, which may inform future studies and public health planning.

## Conclusions

The contemporary prevalence of OAB was high, affecting 14.5% US men. The overall prevalence of OAB significantly increased across recent two decades, especially among men who were aged 40–59 years, non-Hispanic White and Black, and overweight and obese men. Future studies are needed to address OAB disparities across sociodemographic subgroups and to investigate the factors driving the rising trends in subtypes of OAB among older men, non-Hispanic White and Black, and obese men. Focused research can help prevent and remedy this growing socioeconomic and individually troublesome malady.

## Data Availability

The datasets generated and/or analyzed during the current study are available in the open database NHANES website: https://wwwn.cdc.gov/nchs/nhanes/Default.aspx.
